# Bioluminescent imaging of *Trypanosoma cruzi* infection in *Rhodnius prolixus*

**DOI:** 10.1186/1756-3305-5-214

**Published:** 2012-09-26

**Authors:** Cristina Henriques, Daniele P Castro, Leonardo HF Gomes, Eloi S Garcia, Wanderley de Souza

**Affiliations:** 1 Laboratório de Ultraestrutura Celular Hertha Meyer, UFRJ, Rio de Janeiro, RJ, Brazil; 2 Laboratório de Bioquímica e Fisiologia de Insetos, Instituto Oswaldo Cruz (Fiocruz), Rio de Janeiro, RJ, Brazil; 3 Laboratório de Genômica Funcional e Bioinformática, Instituto Oswaldo Cruz (Fiocruz), Rio de Janeiro, RJ, Brazil; 4 Instituto Nacional de Metrologia, Qualidade e Tecnologia, Inmetro, Duque de Caxias Xerém, RJ, Brazil

**Keywords:** *Trypanosoma cruzi*, *Rhodnius prolixus*, Chagas diseases, Bioluminescent imaging, Bioluminescence, Luciferase

## Abstract

**Background:**

Usually the analysis of the various developmental stages of *Trypanosoma cruzi* in the experimentally infected vertebrate and invertebrate hosts is based on the morphological observations of tissue fragments from animals and insects. The development of techniques that allow the imaging of animals infected with parasites expressing luciferase open up possibilities to follow the fate of bioluminescent parasites in infected vectors.

**Methods:**

D-luciferin (60 μg) was injected into the hemocoel of the whole insect before bioluminescence acquisition. In dissected insects, the whole gut was incubated with D-luciferin in PBS (300 μg/ml) for *ex vivo* bioluminescence acquisition in the IVIS® Imaging System, Xenogen.

**Results:**

Herein, we describe the results obtained with the luciferase gene integrated into the genome of the Dm28c clone of *T. cruzi*, and the use of these parasites to follow, in real time, the infection of the insect vector *Rhodnius prolixus*, by a non- invasive method. The insects were evaluated by *in vivo* bioluminescent imaging on the feeding day, and on the 7 th, 14 th, 21 st and 28 th days after feeding. To corroborate the bioluminescent imaging made *in vivo,* and investigate the digestive tract region, the insects were dissected. The bioluminescence emitted was proportional to the number of protozoans in regions of the gut. The same digestive tracts were also macerated to count the parasites in distinct morphological stages with an optical microscope, and for bioluminescence acquisition in a microplate using the IVIS® Imaging System. A positive correlation of parasite numbers and bioluminescence in the microplate was obtained.

**Conclusions:**

This is the first report of bioluminescent imaging in *Rhodnius prolixus* infected with trypomastigotes of the Dm28c-luc stable strain, expressing firefly luciferase. In spite of the distribution limitations of the substrate (D-luciferin) in the insect body, longitudinal evaluation of infected insects by bioluminescent imaging is a valuable tool. Bioluminescent imaging of the digestive tract infected with Dm28c-luc is highly sensitive and accurate method to track the fate of the parasite in the vector, in the crop, intestine and rectum. This methodology is useful to gain a better understanding of the parasite – insect vector interactions.

## Background

Chagas disease is an endemic parasitic disease that is ranked as one of the most important in several areas of Latin America. Triatomine bugs are vectors of the parasite *Trypanosoma cruzi*, the causative agent of Chagas disease. The gut of the vector *Rhodnius prolixus* is basically comprised of three major regions, the foregut (stomach or crop), the midgut (intestine), and the hindgut (rectum); however more elaborate divisions have been proposed [1,2]. These different regions are important for the development of the parasite [3,4]. The vector host digestion starts when the insect ingests blood from the vertebrate host that is then stored in the crop where it is concentrated and hemolyzed. Subsequently, it passes into the intestine for digestion and absorption, followed by storage of the non absorbed products in the rectum and subsequent release. The remaining compounds are swept by the urine and feces that are secreted rapidly after new blood ingestion [2,5].

*T. cruzi* development begins inside the insect vector as soon as the insect has fed on the blood of an infected host. In the crop, most of the trypomastigotes differentiate into epimastigotes, the main replicative stage. A significant proportion of parasites are lysed in part due to the interaction with bacteria found in the crop microbiota [6]. Subsequently the remaining epimastigotes migrate to the intestine where they proliferate and adhere to the perimicrovillar membranes. Then the epimastigotes transform into the non-replicative, but infective metacyclic trypomastigotes, which are released in the urine and feces [2,5,7,8].

Until now the study of the distribution of *T. cruzi* inside the insect vector has been based on the microscopic examination of the digestive tract following dissection of the insects. However, recently the use of bioluminescent imaging of the whole animal has shown that it is possible to follow the parasite *in vivo* within their mammalian hosts if the parasites are labeled with luciferase [9,10]. In the present work we describe the results obtained with the integration of the luciferase gene in the genome of the Dm28c clone of *T. cruzi*, and the use of the labeled parasites to follow, in real time, the infection of the invertebrate vector *R. prolixus*. We observed the infected insects for one month after feeding/infection, and the light emitted could be traced continuously within the insect. We also analyzed insects that were dissected to expose the digestive tract. This methodology will be useful for further studies to acquire a better understanding of the parasite – insect vector interactions.

## Methods

### Expression of firefly luciferase (Fluc) *in Trypanosoma cruzi*

The luciferase gene was amplified by PCR using specific primers. The forward primer contains an XbaI site and the Kozak sequence (underlined), upstream of the start codon, 5′- GCTCTAGA GCCACC ATGGAAGACGCCAAAAACATAAAG – 3′ (F-luc), and the reverse primer contains an XhoI site (underlined) 5′- CCGCTCGAG CGGTTACACGGCGATCTTTCC- 3′ (R-luc). Amplification was carried out using the Tli DNA Polymerase (Promega) and the following PCR conditions: 94^o^C, 5 min; 94^o^C, 30 sec; 60^o^C, 30 sec; 72^o^C, 2 min, 30 cycles; 72^o^C, 10 min. The PCR product was cloned into the Zero Blunt TOPO PCR Cloning Kit (Invitrogen), digested from the TOPO vector and subcloned into the integrative pTREX vector at the XbaI and XhoI restriction sites [11]. The construction was sequenced on an ABI 3730 Genetic Analyzer (Applied Biosystems), using the sequencing platform form PDTIS/FIOCRUZ.

*T. cruzi* epimastigotes of Dm28c clone were suspended at 1 x 10^8^ cells/mL in electroporation buffer (EPB) containing 137 mM NaCl; 5 mM KCl; 0.7 mM Na_2_HPO_4_; 6 mM glucose; 21 mM HEPES, pH 7.3. The cellular suspension (400 μl) was mixed with 50 μg of plasmid, digested with NheI, placed in a 0.2 cm cuvette and subjected to a pulse of 0.45 kV, 500 μF at room temperature in a Gene Pulser apparatus (BioRad Laboratories) [12]. The parasites were re-suspended in LIT medium and stable transformants were selected with 200 to 500 μg/mL of G418. Thereafter, the high expressing epimastigotes were selected by serial dilution in a 96 well plate, and selected by bioluminescent emission with Steady-Glo reagent (Promega) in a microplate reader SpectraMax2.

### Genomic southern blot

To examine the integration of the Fluc gene and the pTREX construction in the *T. cruzi* genome we performed Southern blot of the epimastigote genomic DNA. Epimastigotes (10^8^) were lysed with 1 ml of buffer (10 mM Tris–HCl, pH7.5, 100 mM NaCl, 0.5% SDS 25 mM EDTA and 0.1 mg/ml of proteinase K). The DNA was isolated by phenol:chloroform extraction and recovered by ethanol precipitation. Genomic DNA from the wild type Dm28c and from the genetically modified Dm28c-luc strain were digested with *Eco*RI restriction enzyme, using the standard protocol, then the restriction fragments were separated by electrophoresis in agarose gel and transferred to a positively charged nylon membrane with a high salt buffer.

To produce the probe, pTREX-luc plasmid was digested with NheI and XhoI. A fragment of 2.2 kb, containing the *neo* gene and the *gapdh* intergenic region, was agarose gel purified and 1 μg was used as template to produce the probe with ready-to-go DNA labeling beads (GE Healthcare). Unlabeled dCTP was added to the reaction mixture according to the manufacturer’s protocol, and incubated for 24 hours. The length of the probe ranged from 200 to 1000 bp; unincorporated nucleotides and reaction buffer were removed by the Wizard® SV Gel and PCR Clean-Up System (Promega). The probe was labeled with alkaline phosphatase by the Gene Images AlkPhos Direct Labeling and Detection System (GE Healthcare) and 50 ng of labeled probe was added to 30 ml of hybridization buffer in a bottle containing the nylon membrane with immobilized DNA, and was then incubated in a hybridization oven for 48 hours at 55^o^C. After hybridization the blots were washed following the manufacturer recommendations. The washed blots were placed directly into the detection system protocol, using the CDP-Star chemiluminescent detection reagent, which uses the probe-bound alkaline phosphatase to catalyze the decomposition of a stabilized dioxetane substrate (GE Healthcare). Autoradiography films were exposed from 10 minutes to 2 hours.

### PCR and sequencing to identify the pTREX-luc integration

To identify the integration of the pTREX-luc construction in the genome, a couple of primers were designed to align the transcription start point, tsp1, of the ribosomal promoter from pTREX, F-TSP1- 5′TCATGGAGCGGTATTCTC-3′ and R-TSP1 5′GAGAATACCGCTCCATGA-3, and another pair of primers to align the ribosomal locus recombination site [13], F-RS pTREX- 5′-GTCCGAACGCGGAAATGT-3′ and R-RS pTREX- 5′-ACATTTCCGCGTTCGGAC-3′. PCR amplifications were performed using genomic DNA from Dm28c-luc as a template and a set of primers: 1- F-TSP1/R-LUC; 2- F-RS pTREX/R-LUC and; F-LUC/R-LUC using the following settings: 94^o^C, 2 min; 94^o^C, 30 sec; 60^o^C, 30 sec; 68^o^C, 2 min, 30 cycles; 72^o^C, 10 min and the Platinum Taq DNA Polymerase High Fidelity (Invitrogen). The PCR fragments were resolved by 1% agarose gel in Tris-Acetate- EDTA (TAE) buffer, stained with ethidium bromide and gel purified by the Wizard® SV Gel and PCR Clean-Up System (Promega). The PCR products were sequenced with primers specific for the ribosomal promoter region: F-TSP1; R-TSP1; F-RS pTREX; R-RS pTREX, on an ABI 3730 Genetic Analyzer (Applied Biosystems), using the sequencing platform from PDTIS/FIOCRUZ.

### Parasite cultivation

Genetically modified epimastigotes of *T. cruzi* (Dm28c-luc), expressing luciferase, and wild type (Dm28c clone) were cultivated in liver infusion tryptose (LIT) medium with 10% fetal calf serum, at 28^o^C until the logarithmic stage of growth [14]. Epimastigotes of Dm28c-luc were cultivated in the same medium, but supplemented with G418 200 μg/ml. The non-infective and replicative epimastigotes were transformed into non-dividing and infective metacyclic trypomastigotes. This is a process known as metacyclogenesis, which is carried out by subjecting *T. cruzi* epimastigotes from the late exponential growth phase at a cell density of 3 x 10^7^ cells/ml to nutritional stress in a triatomine artificial urine (TAU) medium (190 mM NaCl; 8 mM phosphate buffer, pH 6.0; 17 mM KCl; 2 mM MgCl_2_; 2mM CaCl_2_) for 2 hours. Then, a further incubation in TAU supplemented with amino acids and glucose (TAU3AAG) (TAU supplemented with 0.035% sodium bicarbonate, 10 mM L-proline, 50 mM sodium glutamate, 2 mM sodium L-aspartate, and 10 mM glucose) [15]. Metacyclic parasites were used to infect the host cell LLCMK2, and trypomastigotes from the cell culture were used to infect the insects.

### Insect infection

Fifth-instar *Rhodnius prolixus* larvae obtained from our colony were used throughout these studies. After molting, insects that had been starved for 15-20 days and weighed 35.2 ± 3.4 mg, were randomly chosen and then allowed to feed on defibrinated rabbit blood through a membrane feeding apparatus [4]. Defibrinated rabbit blood used for feeding the insects was provided by the Laboratory Animals Creation Center of Fiocruz (Cecal). All research programs using Cecal respect the guidelines of the Ethics Committee on Animal Use (Ceua) composed of Fiocruz researchers and external consultants. A control group was fed with blood alone and infected groups on blood containing ~1 x 10^7^ tissue culture-derived trypomastigotes of *T. cruzi* Dm28c clone per ml of blood meal. The experimental group was fed with blood infected with genetically modified trypomastigotes, Dm28 luc expressing luciferase, at 1.7 x 10^7^ trypomastigotes per ml of blood. Only fully gorged insects were used (180.5 ± 22.1 mg) and partially fed insects were discarded. All insects were raised and maintained as previously described [4]. To analyze the insect infection on the feeding day, D-luciferin substrate was given at 1 mg/mL by oral treatment together with the bloodmeal containing the wild type Dm28c or the genetically modified Dm28c-luc trypomastigotes and imaged in the IVIS® Lumina Imaging System at the Bioimaging Central Unit/National Institute of Science and the National Institute of Science and Technology for Structural Biology and Bioimaging INBEB/CENABIOII/UFRJ.

### Substrate treatment

For bioluminescent imaging on days 7, 14, 21 and 28 after feeding, the insects were inoculated laterally in the thorax with 2 μl of D-luciferin solution (30 mg/ml) using a 30 gauge hypodermic needle (BD Precision Glide) adapted to a 10-μl Hamilton syringe. Afterwards, the insects were immobilized by attaching the dorsal region with a double-sided adhesive tape and after 5 min they were put into the IVIS® Lumina Xenogen equipment to acquire bioluminescence. To avoid mortality through successive inoculations we tested the acquisition of bioluminescent imaging with D-luciferin topical applications. The insects were immobilized as described above and 5 μl of D-luciferin solution (30 mg/ml) was applied over the insect ventral region. The insects were kept immobilized for 15 min to let the compound penetrate through the cuticle before capturing the bioluminescence. To analyze the parasite migration into the digestive tract, the insects were dissected and the organ was incubated with D-luciferin, 300 μg/mL in PBS, for 5 min in Petri dishes. The digestive tracts were then analyzed with the bioluminescence equipment, IVIS® Lumina Xenogen, at the Bioimaging Central Unit of the National Institute of Science and the National Institute of Science and Technology for Structural Biology and Bioimaging INBEB/CENABIOII/UFRJ .

### *In vivo* and *ex vivo* bioluminescent imaging

*In vivo* bioluminescent imaging was done with fifth-instar *R. prolixus* larvae infected with *T. cruzi,* that after treatment with D-luciferin were evaluated on the feeding day and on days 7, 14, 21 and 28 after feeding in the bioluminescence image system (IVIS 100; Xenogen, Alameda, CA). The IVIS® Lumina, Xenogen is composed of a highly sensitive CCD camera, a light-tight imaging chamber with complete computer automation, and the Living Image® software for image acquisition and analysis. To acquire the bioluminescent images of the low level of luminescence emissions in the insects and their digestive tracts, parameters such as the time of exposure, which varied from some seconds until 5 minutes, the size of pixel represented by the binning medium and the field of view (FOV) in the B position, 7.5 cm, with automatic focus were used. The Living Image software automatically co-registers the luminescent image, taken in darkness and displayed in pseudo-colors that represent the intensity of the signal, and the photographic image to generate an *overlay* image.

To confirm the bioluminescent imaging made *in vivo* and to evaluate the digestive tract of the infected insect on different days of the development cycle, the insects were dissected and the whole gut removed. The digestive tract, *ex vivo*, was incubated with D-luciferin and the luminescence acquired for a few seconds to 5 minutes in the IVIS® Lumina from Xenogen. To correlate the luminescent emissions and the number of parasites, the whole digestive tract was homogenized in 1ml of sterile phosphate saline buffer (PBS) and the parasites counted in a Neubauer chamber. Afterwards, macerated digestive tracts were centrifuged at 2200 g for 10 min, the pellet was re-suspended in 200 μl of PBS containing D-luciferin 300 μg/ml and then placed into the 24 wells microplate, which was imaged for bioluminescence in the IVIS® Lumina Xenogen, at the Bioimaging Central Unit of the National Institute of Science and the National Institute of Science and Technology for Structural Biology and Bioimaging INBEB/CENABIOII/UFRJ.

### Image analysis

Two experiments were done with 129 and 46 insects. To measure the photon radiance, regions of interest (ROI) were selected on the surface of the insect with the automatic ROI tool and the bioluminescence was measured quantitatively by the Living Image® software, which gave the total flux of photons or radiance (photons/second from the surface) in each pixel summed or integrated over the ROI area, in a square centimeter (cm^2^) of the tissue, multiplied by one steradian (sr). The photon radiance is displayed as the average radiance, which is the sum of the radiance from each pixel inside the ROI/number of pixels or super pixels (photons/sec/cm^2^/sr) and the standard deviation of the pixel radiance inside the ROI. The correlation coefficient was calculated by linear regression of the average radiance versus the number of parasites counted in the Neubauer chamber. The results are presented as the average and standard error of photon radiance.

## Results and discussion

### Expression of luciferase *in Trypanosoma cruzi*

**Figure 1:**
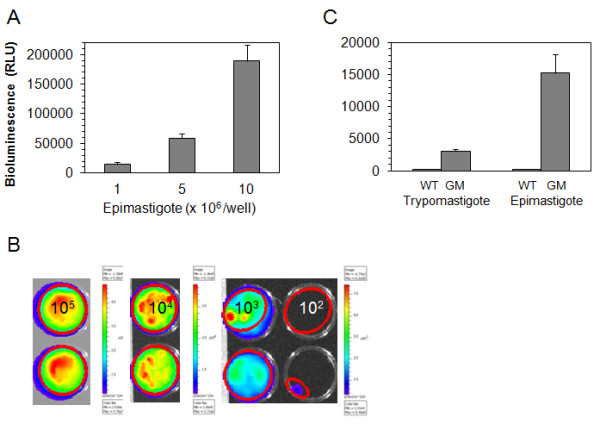
**Bioluminescence from genetically modified strain, Dm28c-luc.** Firefly luciferase expression in Dm28c-luc epimastigotes was evaluated by bioluminescent emission with increasing number of parasites per well, on the microplate reader with SteadyGlo reagent (**A**), and by the CCD camera of the Xenogen imaging system, in the 24 well plate with D-luciferin in PBS (**B**). Comparison of epimastigote and trypomastigote bioluminescent emissions were made on the microplate reader, using SteadyGlo reagent and 10^6^ parasites per well, Dm28c wild type (WT) and Dm28c-luc (GM) (**C**).

Stable transfectant epimastigotes of the Dm28 strain expressing luciferase were produced by electroporation of the pTREX/luc linear plasmid, and selected with Geneticin (G418) followed by cloning by serial dilution. The genetically modified bioluminescent epimastigotes were stable after two years of cultivation in medium with 200 μg/ml of G418 and bioluminescence was proportional to the number of *T.cruzi* expressing luciferase (Figure 1 A and B, Table 1). The Dm28c-luc epimastigotes displayed six fold higher bioluminescence (15.2 ± 2.9, n = 3) relative luminescence unit (RLU) (Figure 1 A) compared to the Brazil clone epimastigotes [10].

**Table 1:** Bioluminescent emission from 24 well plate

**Epimastigotes/well**	**Avg Radiance (p/s/cm**^**2**^**/sr)**	**Stdev Radiance**
10^5^	1.8 x 10^10^	5.2 x 10^9^
10^4^	9.3 x 10^8^	2.4 x 10^8^
10^3^	2.6 x 10^8^	1.1 x 10^8^
10^2^	1.8 x 10^7^	9.8 x 10^6^

Bioluminescence of epimastigotes was performed in a microplate with D-luciferin 300 μg/ml in PBS, acquired by the CCD camera, and quantified by the Living Image® software with the ROI measurement tool.

**Figure 2:**
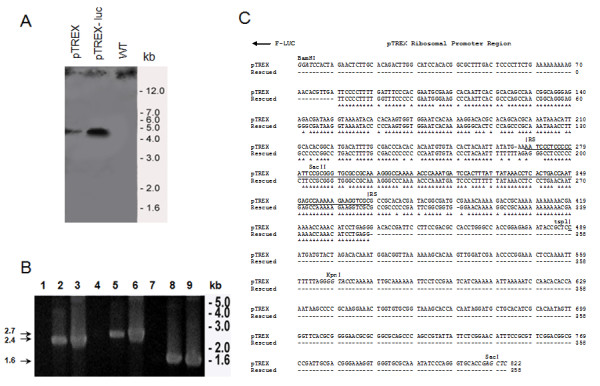
**Integration of pTREX-luc into the Dm28c genome.** Southern blot of epimastigote genomic DNA from Dm28c stably transfected with pTREX, cloned Dm28c-luc, transfected with pTREX-luc, and Dm28c wild type were digested with EcoRI and probed with *neo/gapdh* sequence (**A**). PCR amplification of ribosomal promoter regions followed by the HX1 and luciferase sequences was performed in genomic DNA from Dm28c wild type (lanes 1, 4 and 7), as negative control; from cloned Dm28c-luc, (lanes 2, 5 and 8); and DNA from pTREX-luc plasmid, (lanes 3, 6 and 9), as positive control, using the specific primers: F-TSP1/R-LUC (lanes 1, 2 and 3) and; F-RS pTREX/R-LUC (lanes 4, 5 and 6). The luciferase gene was amplified by PCR, with F-LUC/R-LUC pair of primers (lanes 7, 8 and 9) (n = 3). All the PCR fragments were evaluated in an agarose gel and the fragments of 2.4 and 2.7 kb, amplified from Dm28-luc genomic DNA (lanes 2 and 5), were gel purified (**B**). The rescued PCR fragments of 2.4 and 2.7 kb and the pTREX-luc plasmid were sequenced with primers: F-TSP1; R-TSP1; F-RS pTREX and R-RS pTREX (n = 2). Ribosomal promoter sequences from rescued PCR fragments and from the pTREX-luc plasmid were aligned with the clustalw 2.1 multiple sequence alignment (**C**). Asterisks correspond to the identical region between the ribosomal promoter of Dm28c-luc strain and the pTREX-luc plasmid; TSP, transcription start point; RS, recombination site, underlined; BamHI, KpnI and SacII, restriction enzymes in the pTREX-luc plasmid.

Trypomastigotes obtained from LLCMK2 cells and cultivated without G418 were stable for 6 months, and displayed a lower expression compared to the epimastigotes (Figure 1 C), as evaluated by bioluminescent emission. Dm28c-luc can probably be stable longer than the period tested, but to avoid selection and changes in the expression of luciferase from the original population, it is recommended to start new cultures from frozen trypomastigotes periodically or metacyclogenesis from frozen stocks of epimastigotes. To investigate the pTREX-luc plasmid integration, Southern blots of genomic DNA from Dm28c transfected with pTREX, genomic DNA of cloned Dm28c-luc and of wild type Dm28c were digested with EcoRI and probed with *neo/gapdh* intergenic region. A faint band was observed in the pTREX uncloned transfectants when compared to pTREX-luc cloned transfectants, which displayed a band of approximately 5 kb (Figure 2 A), correspondent to a fragment of the genome cleaved by EcoRI enzyme, a restriction site inexistent in pTREX-luc plasmid.

To evaluate the integration of pTEX-luc, PCR of genomic DNA was performed using specific primers to amplify regions of the ribosomal promoter, followed by the luciferase gene. Single PCR products were amplified from genomic DNA of cloned Dm28c-luc, generating fragments of expected size as compared with PCR amplified fragments of pTREX-luc plasmid (Figure 2 B). The PCR of wild type Dm28c genomic DNA was negative for the pairs of primers tested (Figure 2 B). Fragments of 2.4 kb and 2.7 kb were amplified by PCR with two sets of primers specific for the ribosomal promoter sequences coupled to the luciferase gene, and a PCR product of 1.6 kb was amplified with primers specific for the luciferase gene (Figure 2 B). The PCR products of 2.4 kb and 2.7 kb, purified from agarose gel, were sequenced and the site of recombination (RS) in the ribosomal promoter, proposed previously as the integration locus in the genome [13], displayed 87 % identity to the ribosomal promoter sequence in the pTREX-luc plasmid, as indicated (Figure 2 C).

### Genetically modified Dm28c-luc versus wild type Dm28c strain

A comparison between the *Rhodnius prolixus* vector infected with *T. cruzi* Dm28c wild type and Dm28c-luc genetically modified was carried out to evaluate the capacity of the genetically modified strain to infect and maintain the infection. Thus, both groups of infected insects were subjected to the same procedure of feeding. Insects infected with Dm28c wild type ingested also D-luciferin, as a negative control of bioluminescent imaging (not shown) and as a control of infection. They displayed similar mortality rates of 24% and 26%, respectively. The percentage of insects infected with Dm28c-luc was 51%, 21 days (n = 35) after feeding, as evaluated by bioluminescent emission, and ranged from 50% to 47%, 21 days (n = 10) and 28 days (n = 19) post infection, as evaluated by parasite counting in dissected digestive tracts. Insects infected with Dm28c wild type displayed 40% infection, as evaluated after dissection and parasite counting 14 days (n = 10) and 21 days (n = 5) after feeding.

In both groups, we observed epimastigotes, spheromastigotes and metacyclic trypomastigotes in the macerated tissues of the digestive tract, which displayed a median of 2.5 x 10^4^ parasites (from 10^3^ to 3.6 x 10^5^ parasites per insect, n = 7) for Dm28c-luc and a median of 3 x 10^4^ parasites (from 2 x 10^3^ to 3.7 x 10^5^ parasites per insect, n = 4) for the Dm28c wild type infected insects, that was maintained for twenty one days of follow up. These values show that Dm28c wild type strain and Dm28c-luc genetically modified have similar infection and proliferative rates in the gut of *Rhodnius prolixus.*

### Insect immobilization and evaluation of substrate delivery method

**Figure 3:**
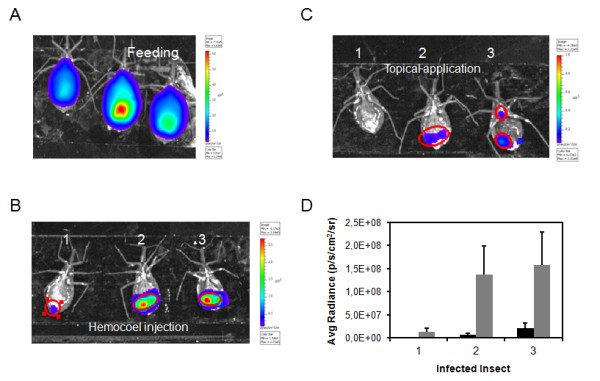
**Substrate delivery method in *Rhodnius prolixus *infected with Dm28c-luc.** On the feeding day, insects ingested an average of 250 μl of blood with trypomastigotes and supplemented with D-luciferin (1 mg/ml), images were obtained after 10 seconds exposure by the CCD camera with binning factor of 2 (**A**). After feeding, insects (1, 2 and 3) were evaluated after 60 μg of D-luciferin was injected into the hemocoel, directly in the hemolymph (**B**); and the same insects were evaluated after 150 μg D-luciferin application to the cuticle, onto the insect ventral thorax, followed by 15 minutes incubation to allow the substrate to diffuse throughout the body (**C**). In both cases, the bioluminescent images were acquired after 5 minutes exposure by the CCD camera and binning factor 4. The bioluminescent spots were selected in the image with the ROI measurement tool and the radiance quantified by Living Image® software (**D**), (gray square) hemocoel injection (black square) topical application.

To acquire bioluminescent images after feeding, the substrate D-luciferin has to be delivered to the insect, immediately before imaging (Figure 3 A). The full activity of the firefly luciferase is reached at 37^o^C, the platform temperature of the IVIS lumina system. Thus, insects immobilized on ice displayed lower bioluminescent emission, as a consequence of reduced luciferase activity. Therefore, it is recommended to immobilize them using double face tape or another restraint method.

Two methods of D-luciferin delivery were tested: injection into the hemocoel/hemolymph and topical application. Substrate injection in the hemolymph is used as a routine for applying drugs and compounds. D-luciferin injetion is fast and the insects can be evaluated by bioluminescent imaging after 5 minutes (Figure 3 B), however, it increases the chance that the insects die with successive cuticle damage. Nevertheless the D-luciferin topical application is an excellent tool to administer the compound to the insect without cuticle damage, especially if it is important to keep the insect alive for several days to acquire more bioluminescence data. This delivery method requires more substrate and prolonged incubation (15 minutes) to allow D-luciferin to penetrate the cuticle and then reach the digestive tract (Figure 3 C). In spite of this, both methods of D-luciferin delivery show limitations related to the distribution of D-luciferin throughout the insect body, but the topical application can be more prone to produce false negatives (Figure 3 D). Thus, the injection of D-luciferin into the hemolymph was the method of choice to perform the bioluminescence evaluation and radiance quantification in this work.

Firefly luciferase (luc), one of the most common bioluminescent proteins employed for *in vivo* studies, requires the injection of the substrate D-luciferin to produce bioluminescent signals. D-luciferin can only produce light upon oxidation and the route of substrate injection can have influential effects on the emission of the bioluminescent signal. Small mammal models have shown that, depending on the method of injection, intraperitoneal, subcutaneous or intravenous injection, the absorption rate of the substrate and distribution throughout the tissues can lead to variations in the bioluminescent signal and affect reproducibility [16]. Studies using radio-labeled D-luciferin injected intravenously demonstrated that the uptake rate of the substrate is actually slower in gastrointestinal organs, pancreas, and spleen than would be achieved using intraperitoneal injection [17]. However, the intravenous injection of the substrate generates a faster bioluminescent signal but with a shorter duration than the intraperitoneal route [18,19]. Thus, the injection method should be considered in light of the proposed objectives of any study.

### Bioluminescent imaging after feeding

A bioluminescent image of *Rhodnius prolixus* infected with *T. cruzi* expressing luciferase acquired in an IVIS® Imaging System is a diffuse projection on the surface of the insect from the trypanosome inside the digestive tract. The bioluminescent spots on the insect are associated with foci of infection along the digestive tract, and the diffusion of D-luciferin.

In our model of study, starved insects were fed with blood meal containing trypomastigotes of Dm28c-luc mixed with D-luciferin. The insects were loaded with trypomastigotes, they ingested an average of 250 to 300 μl of blood with trypomastigotes, which represents 4.3 - 5.1 x 10^6^ trypomastigotes per insect (Figure 3 A). In this particular situation the emission of photons should be proportional to the amount of trypomastigotes swallowed (Figure 3 A, Table 2). Thus, the high flux of photons from the insects replete with blood filling the hemocoel was sufficient to saturate the IVIS® Imaging System after 30 seconds. Therefore, the images were acquired with just a 5 to 10 seconds exposure (Figure 3 A). The images were taken immediately after the blood meal, which lasted approximately 30 minutes. In the control insects, which were fed with blood containing wild type Dm28c strain and the substrate D-luciferin, bioluminescence was absent even after 5 min exposure by the CCD camera (not shown).

**Table 2:** **Bioluminescent emission in the insect and dissected digestive tracts of *Rhodnius prolixus *infected with Dm28c-luc**

**Bioluminescence Days after feeding**	**Insect Radiance (p/s/cm**^**2**^**/sr)**			**Dissected Digestive Tract Radiance (p/s/cm**^**2**^**/sr)**		
	**Median**	**Min**	**Max**	**Media**	**Min**	**Max**
Feeding	**2.1x 10**^**11**^	9.1 x 10^10^	4.8 x 10^11^	ND	ND	ND
7	**1.8 x 10**^**8**^	6.9 x 10^5^	4.9 x 10^9^	**1.6 x 10**^**9**^	1.3 x 10^8^	3.3 x 10^9^
14	**8.8 x 10**^**7**^	4.2 x 10^6^	7.9 x 10^9^	**9.0 x 10**^**8**^	1.8 x 10^7^	6.9 x 10^9^
21	**3.9 x 10**^**7**^	3.8 x 10^6^	2.8 x 10^9^	**4.0 x 10**^**8**^	6.3 x 10^7^	2.9 x 10^9^
28	**6.9 x 10**^**7**^	1.4 x 10^7^	2.7 x 10^8^	**1.3 x 10**^**9**^	1.8 x 10^7^	4.8 x 10^9^

The automatic ROI tool was used to select the region used to estimate the photon radiance emitted from a group of insects and from the dissected digestive tracts on the feeding day and on days 7, 14, 21 and 28 after feeding. The data is displayed as median radiance, with minimum and maximum radiance, from each group of insects used in the analysis. Not determined (ND).

### Bioluminescent imaging

In experiments using several insects, it is worth noting that insects with high photon emission can mask the spots of insects with low photos emission, obtained by the CCD camera. Bioluminescent imaging in Living Image software has a minimum threshold, the image is an overlay of a luminescent image over a grayscale photographic image, the upper (Max Bar) and lower limits (Min Bar) are in the color table display. All photon emissions below the Min Bar setting of relatively lower bioluminescent emission are not displayed in the pseudocolor image, and are transparent, to avoid saturation of the image.

The pseudocolor scheme makes it easy to quantify spot regions of bright light emission, but because the range of radiance in our model of study varied from 5 x 10^9^ to 6.9 x 10^5^ photons/sec/cm^2^/sr (Table 3), it is highly recommended to inspect negative insects, by removing the insects which display higher measurements of radiance and make a new bioluminescence acquisition, until bioluminescence is not detected. The sensitivity of lower-photon emitter insects is dependent on the relationship of the number of photons versus background, or noise of the image. The software automatically computes a default minimum radiance value (Min Bar) and this is the recommended starting point for an analysis.

**Table 3:** Insect classification by bioluminescent emission

**Bioluminescence 7 days after feeding**	**Insect Radiance (p/s/cm**^**2**^**/sr)**		
	**Median**	**Min**	**Max**
Super High (n = 8)	2.5 x 10^9^	1 x 10^9^	4.9 x 10^9^
High (n = 30)	3.2 x 10^8^	1 x 10^8^	8.8 x 10^8^
Medium (n = 18)	3.6 x 10^7^	1.4 x 10^7^	9.8 x 10^7^
Low (n = 8)	5.1 x 10^6^	6.9 x 10^5^	9.1 x 10^6^

Seven days after feeding, all insects were evaluated by bioluminescent emission *in vivo,* after D-luciferin injection into the hemocoel, and divided in four groups based on the photon radiance measured in the selected region of interest (ROI).

In our study the IVIS® Imaging System was set to acquire photons for 30 seconds to 5 minutes exposure, in the *ex-vivo* assay, and for 5 minutes, in the whole insect. To acquire all the information from the photons emitted by the whole insect and their tissues infected with *T. cruzi* Dm28c-luc, other parameters such as binning were considered. Therefore, after imaging a group of insects with high luminescence it is important to set up the equipment again to obtain images of insects with lower bioluminescence. If the luminescence does not appear it is recommended to increase the exposure time to 5 min before considering the insect negative. In addition, it is also recommendable to dissect the insect and make an *ex vivo* bioluminescent imaging.

### Bioluminescent imaging 7 days after feeding: *in vivo* versus *ex vivo* evaluation

**Figure 4:**
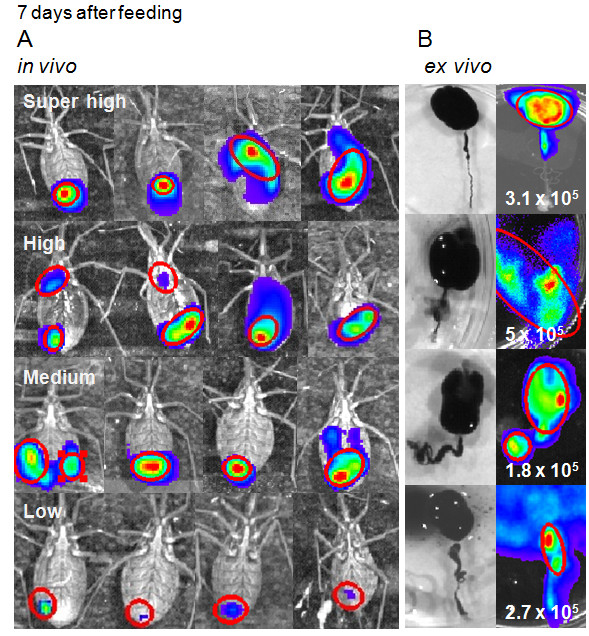
**Bioluminescent imaging of *Rhodnius prolixus *infected with Dm28c-luc.** On the seventh day after feeding, all insects were evaluated by bioluminescent emission *in vivo,* after D-luciferin injection into the hemocoel, and divided in four groups based on the radiance measured, that was predominant in the posterior region of the insect (**A**). In contrast, the *ex vivo* bioluminescent imaging from the distensible crop of the dissected digestive tract, displayed intense bioluminescence in the crop, corroborated by parasite quantification in a Neubauer chamber, as displayed in the enlarged images (**B**).

Fully gorged triatomines do not require another blood meal containing D-luciferin for about a month. Therefore, on the seventh day after feeding, all insects were screened for bioluminescent emission by injecting D-luciferin into the hemocoel (in the hemolymph). The insects were divided in positive and negative groups, sixty four positive insects were identified, approximately 55%, which displayed different degrees of bioluminescence/photon emission. However, the bioluminescent emission of the whole insect was predominant in the posterior region of the abdomen. In contrast, in dissected insects the digestive tract displayed intense bioluminescence in the crop and not in the posterior region of the digestive tract (Figures 4 A and B). Epimastigotes were predominant in a group of insects dissected. The parasites were counted in the macerated digestive tract, which displayed a median of 2.1 x 10^5^ parasites (from 10^4^ to 5 x 10^5^ parasites per insect, n = 10) (Figure 4 B). This number of parasites represents 4.5 % (min 0.2% – max 10%) of parasites ingested after feeding, which suggest that most trypomastigotes died and only a small percentage could survive the hostile environment of the crop and transform into epimastigotes.

In the group of positive insects, bioluminescent emission could be classified in four degrees of photon radiance quantified in the bioluminescent spots on the insect surface (Table 3). More than 70% of luminescent insects displayed bioluminescence in the range of high photon radiance (30 insects) and medium photon radiance (18 insects), whose medians were 3.2 x 10^8^ and 3.6 x 10^7^, photons/sec/cm^2^/sr (Table 3) respectively. Some insects were selected from these groups and dissected. The crop, full of digested blood residues mixed with luminescent parasites, is susceptible to disruption during dissection, however, their digestive tracts displayed radiance consistently higher than the whole insect, median of 1.6 x 10^9^ photons/sec/cm^2^/sr (Table 2). We also dissected three negative bioluminescent insects and observed that two were infected and just one was not. Therefore, from the 45 % negative bioluminescent insects a percentage is probably infected but due to D-luciferin diffusion and distribution throughout the insect body, the infection rate evaluated by bioluminescent imaging could be under detected.

### Bioluminescent imaging 14 days after feeding, *in vivo* versus *ex vivo* evaluation

**Figure 5:**
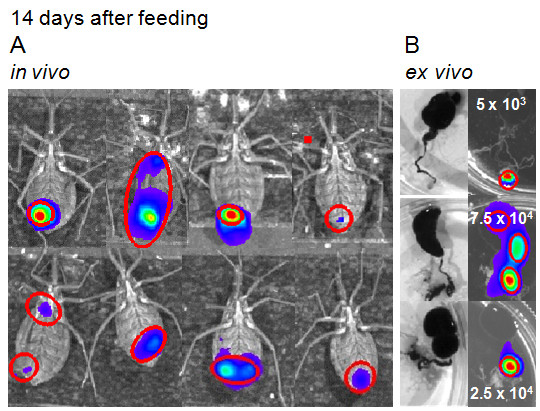
**Life cycle of Dm28c-luc strain in *Rhodnius prolixus*. ***In vivo* (**A**) and *ex vivo* (**B**) evaluation, fourteen days after feeding, displayed the progression of infection. Instead of the crop region, bioluminescence is found in different regions along the intestine (**B**), corroborated by parasite evaluation in macerated digestive tract and quantified in a Neubauer chamber, as displayed in the images (**B**).

Two weeks after feeding, the digestion of the blood meal is close to completion, the crop is no longer bioluminescent for the majority of insects (Figures 5 A and B), epimastigotes and spheromastigotes were found by light microscopy in macerated digestive tracts, some were found attached to portions of the gut epithelium. Metacyclic trypomastigotes were also found in macerated guts and in feces and urine of some insects, in agreement with the bioluminescent imaging of the rectal region of the dissected digestive tract (Figure 5 B).

Photon emissions acquired by the CCD camera did not have enough spatial resolution to show the regions of the intestine and the coiled digestive tract inside the insect. In the insect the bioluminescent image is just a spot that represents part of the infection site. However, the dissected guts can show the precise localization of the parasites along the tract (Figures 5 A and B). In the intestine, scattering and diffusion of light throughout the tissues is not a limiting factor for bioluminescent imaging acquisition. Thus, in dissected insects fast and reliable information can be obtained from infected digestive tract using the CCD camera.

However, it is not always possible to show correlation between the number of parasites in dissected digestive tracts and the bioluminescence analyses in radiance photons/sec/cm^2^/sr. This could be related to the photons emitted from the source inside and along the tract, which will be compact or disperse depending on the amount of pathogens and their distribution throughout the gut (Figure 5 B). Another explanation could be the diversity of parasite life forms encountered inside the gut. Trypomastigote forms obtained from host cells, displayed lower bioluminescence than epimastigote forms (Figure 1 C) which suggest that metacyclic trypanosome is also a lower photon emitter compared to other forms found inside the gut.

### Bioluminescent imaging 21 to 28 days after feeding, *in vivo* versus *ex vivo* evaluation

**Figure 6:**
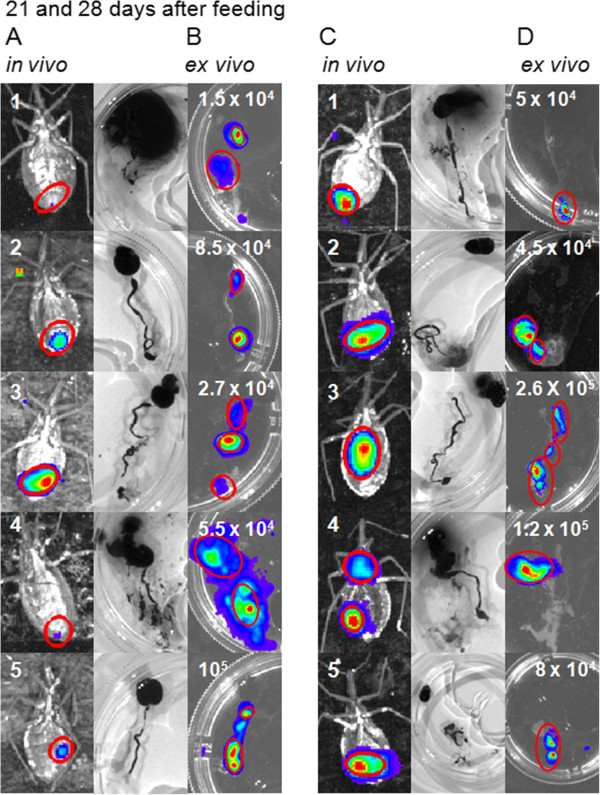
**Life cycle of Dm28c-luc strain in *Rhodnius prolixus*.** Bioluminescent imaging of the insect, after D-luciferin injection in the hemocoel, (insects 1, 2, 3, 4 and 5) and their corresponding dissected digestive tracts, was conducted twenty one to twenty eight days after feeding. *In vivo* (**A**, **C**) and *ex vivo* (**B**, **D**) evaluation, demonstrated variability in the distribution of parasites, along the intestine and the rectum (**C**, **D**). Metacyclic trypomastigotes were observed in feces and urine, but other parasite forms were also observed in the macerated digestive tracts. Parasites were quantified in a Neubauer chamber and displayed in the images (**B**, **D**).

The insects displayed a persistence of the infection for at least one month, when they were all dissected. The insects were evaluated for the presence of infection by injecting D-luciferin in the hemocoel, which produced images of the terminal region of the insects, corresponding to the intestine and rectum (Figures 6 A and C). To corroborate the bioluminescent imaging with the whole insect and observe the parasite mobility throughout the gut, the insects were carefully dissected to avoid disruption of the digestive tract and to maintain the parasites inside the gut region (Figures 6 B and D). Consistent with the assessments made previously, the digestive tract of dissected insects displayed higher radiance than live insects (Table 2).

On the 21st and 28th days after feeding, ten and nineteen insects infected with Dm28c-luc were dissected. Metacyclic trypomastigotes, spheromastigotes and epimastigotes were found in the macerated tissues of the digestive tract. In the third week after feeding, the infection rate was maintained, 50% of insects were infected and the median was 4.1 x 10^4^ parasites (from 1.5 x 10^4^ to 10^5^ parasites per insect, n = 5). After 4 weeks, nineteen insects were evaluated, 47 % of the insects remained infected, the parasites counted in the Neubauer chamber had a median of 10^5^ parasites (from 2 x 10^4^ to 2.7 x 10^5^ parasites per insect, n = 9).

**Figure 7:**
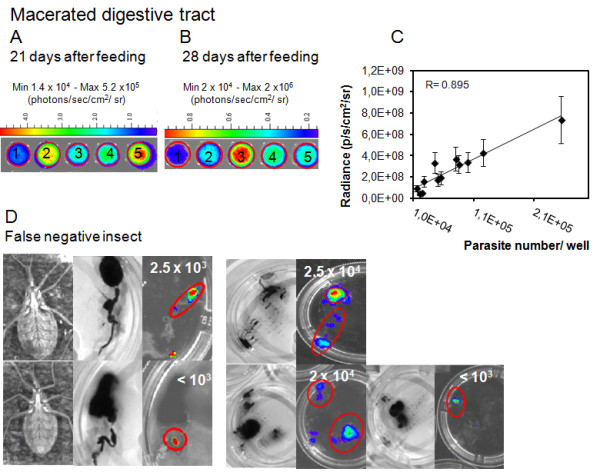
**Evaluation of dissected digestive tracts by bioluminescent imaging.** The whole gut from insects, evaluated in the third and fourth weeks after feeding (named 1, 2, 3, 4 and 5), were macerated. The parasites were counted in a Neubauer chamber and evaluated by the CCD camera in a 24 well plate (**A**, **B**). The photon radiance measured in each well of the plate was positively correlated to the parasite number, R = 0.895 (n = 11) (**C**). False negative insects were observed along the life cycle by bioluminescent imaging, due to D-luciferin diffusion and distribution throughout the insect body, in contrast with the dissected digestive tract (**D**). Parasites were counted in a Neubauer chamber and the numbers are displayed in the images (**D**).

Considering the low correlation between parasite number, quantified in macerated digestive tracts in the Neubauer chamber, with bioluminescent imaging of the living insects or the whole digestive tracts, we used another approach. In the same group of macerated digestive tracts, parasites were counted and were evaluated by bioluminescent emission on the microplate in the IVIS lumina system (Figures 7 A and B). The correlation coefficient was 0.895 (n = 11), which means that the radiance quantified in the plate, but not in the intact intestine and rectum, is proportional to the number of parasites colonizing the digestive tract (Figure 7 C).

On the 21st and 28th days after feeding, a positive correlation between insect bioluminescent imaging and bioluminescence from dissected gut was expected, considering that the infection was more restricted to the terminal regions of the gut. However, due to the distribution of the substrate D-luciferin throughout the insect body, distribution of parasites inside the gut and the conformation of the intestine inside the hemocoel, the coefficient of correlation was low and some insects were false negatives. We observed occasionally that when the insect is colonized with a number of *T. cruzi* below or equivalent to 2.5 x 10^4^ parasites, the insect can be negative but the dissected digestive tract displays the bioluminescence (Figure 7 D). After dissection bioluminescence could be quantified precisely in false negative insects, the median was 2.5 x 10^3^ parasites (from 10^3^ to 2.5 x 10^4^ parasites per insect, n = 5) (Figure 7 D). Thus it is recommended to work with highly infected insects that can be followed up for a month, overcoming the limitations encountered with low parasite load, in infected insects.

### Longitudinal evaluation of labeled insects

**Figure 8:**
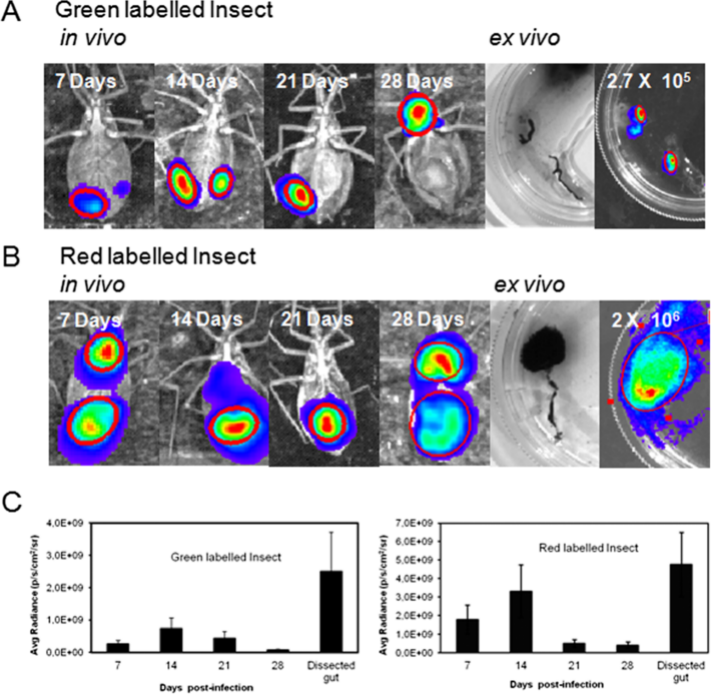
**Longitudinal evaluation of *Rhodnius prolixus *infected with Dm28c-luc.** Evaluation of insect infection by bioluminescent imaging was performed, for a month, *in vivo* with the insects that were labeled with green (**A**) and red (**B**) ink, before dissection. Digestive tract evaluation, from the same infected insects, by *ex vivo* bioluminescent imaging (**A** and **B**). The bioluminescent regions, from insects and digestive tract, were selected in the image by the ROI measurement tool and the radiance quantified by Living Image® software (**C**).

In spite of the substrate diffusion limitation, the same insects can be followed up over several days and the infection can be tracked for a month. Thus, a group of insects were selected according to bioluminescence intensity on the seventh day after feeding, for longitudinal evaluation. These insects were marked with a spot of different colors of water paint (gouache ink), on the dorsal region. Two insects were evaluated for a month by bioluminescence acquisition, before dissection and microscopic counting of parasites in the digestive tract (Figures 8 A and B). However, as discussed before, the digestive tracts displayed higher bioluminescence than the whole insect (Figure 8 C).

**Figure 9:**
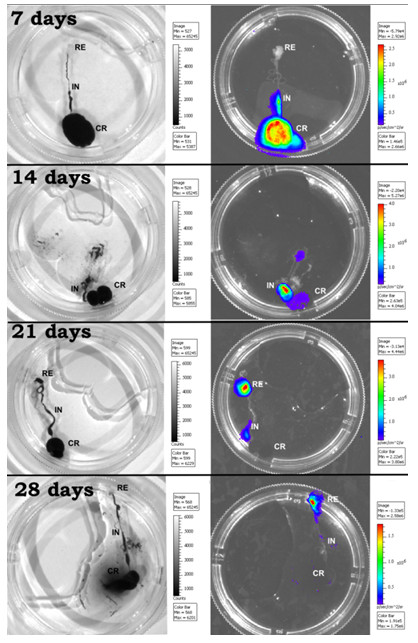
**Bioluminescent imaging of *T.cruzi *in *Rhodnius prolixus *digestive tract.** Dissected insects infected with Dm28c-luc were selected at random 7, 14, 21 and 28 days after feeding to verify the distribution of bioluminescence/parasites infecting regions of the digestive tract. Crop (CR), Intestine (IN), rectum (RE).

To observe the parasite cycle and the progression of infection in the digestive tract, the insects were carefully dissected and the infection could be tracked accurately through the crop, intestine and rectum by bioluminescent imaging (Figure 9). The infection also displayed some variations among the groups of insects.

Previous studies, reported the use of parasites expressing fluorescent proteins to track the fate of the parasite inside the vector. *Rhodnius prolixus* infected with DsRed-labeled *T.cruzi* was used as a marker to follow the parasite life cycle and investigate co-infection with *T. rangeli* expressing GFP [20]. *Plasmodium falciparum* genetically modified lines that stably express gametocyte-specific GFP-luciferase reporters were used as a tool to evaluate the dose- and time-dependent drug action on gametocyte maturation and transmission, evaluated by bioluminescent emission in a plate reader and by light fluorescence microscopy. Mature gametocytes treated with drugs, were formulated as artificial mosquito blood meals and fed to *A. stephensi* mosquitoes. Mosquito midguts were dissected 6 or 7 days after gametocyte ingestion to ascertain the percentage of infected mosquitoes and quantify oocyst production. However, the positive effect of drugs on the inhibition of mature gametocyte transmission to *Anopheles* mosquitoes was not evaluated by *in vivo* or *ex vivo* bioluminescent imaging but by oocyst quantification under a phase-contrast microscope [21].

The fact that dissected digestive tracts display higher bioluminescent emission than the intact insect is probably related to the diffusion of D-luciferin throughout the hemolymph and tissue regions. We can speculate that the substrate D-luciferin (a) is not achieving an adequate concentration in the anterior region of the insect, crop, (b) may be degraded in lipidic bodies, or (c) excreted through Malpighian tubules, involved in elimination of products of digestion, and in ionic and osmotic regulation through the action of channels, exchangers and transporters [22,23]. D-luciferin is transported through the ABCG2/BRCP transporter [24], thus specific transporters and exchangers in the insect organs could increase the turnover of D-luciferin in the hemolymph, and reduce bioluminescent emission in the insect.

## Conclusions

To our knowledge this is the first report of bioluminescent imaging in *Rhodnius prolixus* infected with Dm28c-luc trypomastigotes, expressing firefly luciferase. The strain displayed stable expression, and reproducible data could be acquired, as evaluated by bioluminescent imaging of intact as well as of dissected digestive tracts of *Rhodnius prolixus*, and confirmed by light microscopy observation of the parasites, in the macerated intestines. We can conclude that, bioluminescent imaging analysis offers a considerable quantity of information regarding the parasites movements in the digestive tract as evaluated by dissected guts.

## Competing interests

The authors declare that they have no competing interests.

## Authors’ contributions

The authors ESG and WS idealized the project, contributed to the experimental design and manuscript. CH produced the strain, Dm28-luc, and LDFG made the southern blot and the evaluation of pTREX-luc integration in the genome. DPC contributed significantly with insect physiology and insect handling information. CH and DPC executed bioluminescent imaging, data analysis and manuscript elaboration. All authors read and approved the final version of the manuscript.
